# Unlocking complexity through neutron scattering: Structure and dynamics of protein–polymer conjugates

**DOI:** 10.1002/pro.70137

**Published:** 2025-05-19

**Authors:** Daniela Russo, Frederik Wurm, Jose Teixeira

**Affiliations:** ^1^ Consiglio Nazionale delle Ricerche & Istituto Officina dei Materiali c/o Institut Laue Langevin Grenoble France; ^2^ Sustainable Polymer Chemistry, Department of Molecules and Materials, MESA+ Institute, Faculty of Science and Technology, University of Twente Enschede The Netherlands; ^3^ Laboratoire Léon Brillouin (CEA/CNRS), CEA Saclay Gif‐sur‐Yvette France

**Keywords:** dynamical fluctuation, isotopic deuteration, neutron scattering, protein–polymer conjugates, radius of gyration

## Abstract

Protein–polymer conjugates are engineered to enhance the pharmacokinetics and stability of biopharmaceuticals. This review delves into the structural and dynamic characteristics of protein–polymer conjugates, employing advanced techniques such as neutron scattering, circular dichroism, and fluorescence spectroscopy. Here, we focus on model proteins like maltose‐binding protein (MBP), bovine serum albumin (BSA), and myoglobin (Mb) conjugated with hydrolysable polyphosphoesters (PPEs) to generate fully degradable polymer–protein conjugates. The study underscores the influence of factors such as the polymers' molar mass, grafting density, hydration levels, and deuteration on protein stability, flexibility, and thermal properties. The study demonstrates that the degree of polymerization, solvation properties, and isotopic composition play crucial roles in determining the behavior of protein–polymer complexes. In particular, neutron scattering techniques are invaluable in unraveling the interaction mechanisms, providing valuable insights that can inform the optimization of protein–polymer conjugates for therapeutic applications.

## 1 INTRODUCTION

Protein‐based biopharmaceuticals offer immense therapeutic potential due to their biocompatibility, biodegradability, and functional specificity. However, their inherent limitations, such as rapid proteolytic degradation, renal clearance, and immunogenicity, often hinder their clinical efficacy. To address these challenges, researchers have explored strategies to enhance protein stability, solubility, and circulation time while minimizing immunogenicity. Protein–polymer conjugates offer several advantages over unmodified proteins (Ouyang et al. 2024; Wu et al. 2015). By increasing the size of the protein through conjugation, researchers can prolong blood‐circulation times, leading to reduced administration frequency. This increased size also shields the protein from proteolytic degradation and antibodies, enhancing its stability and reducing immunogenicity (Wright et al. 2019). Additionally, protein–polymer conjugates demonstrate improved bioavailability, transport, resistance, and steric shielding, which can enhance their therapeutic efficacy. Moreover, the conjugation of proteins with polymers can facilitate passive targeting, allowing for increased accumulation in tumor tissues due to the enhanced permeability and retention (EPR) effect. Furthermore, stimuli‐responsive polymers can be incorporated into protein–polymer conjugates, enabling the tuning of protein function on demand, providing greater control over therapeutic outcomes. Therefore, this promising approach implies the creation of protein–polymer conjugates (Duncan 2003; Liu et al. 2018).

**FIGURE 1:**
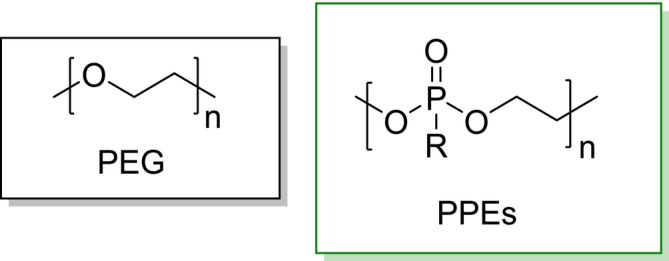
The chemical structures of poyl(ethylene glycol) and PPEs (R typically CH3, CH2CH3, or OCH2CH3).

Traditionally, PEGylation has been the standard for protein conjugation. By attaching polyethylene glycol (PEG) to proteins, researchers can significantly enhance their circulation time, reduce immunogenicity, and improve solubility (Hou and Lu 2019). These advantages have made PEGylation a popular strategy for extending the half‐life of therapeutic proteins. However, the non‐biodegradable nature of PEG, coupled with its potential for triggering hypersensitivity reactions upon repeated administration, has prompted the search for alternative polymers with more favorable properties (Qi and Chilkoti 2015). As synthetic alternative, water‐soluble, biocompatible polymers, including finely tunable derivatives of polyoxazolines, polyacrylates, or polyglycerols, as well as (bio)degradable polyphosphoesters (PPEs), have emerged as promising alternatives for PEG (Figure 1). These polymers offer a diverse range of properties and functionalities, making them promising candidates for various biomedical applications. PPEs can be obtained as hydrophilic, biocompatible, and biodegradable materials with high chemical versatility and following controlled or so‐called “living” syntheses (i.e., low molar mass dispersity, high purity, end‐group fidelity). This class of polyesters is designed to break down either hydrolytically or enzymatically. These unique degradation characteristics allow the conjugated polymer to be gradually released, releasing the protein at its target site, reducing proteolytic degradation and immunogenicity (Steinbach et al. 2014; Steinbach et al. 2017; Steinbach and Wurm 2015; Steinbach and Wurm 2016). PPEs inherently possess a hydroxyl end‐group, which can be used to tether them on proteins or surfaces. The hydroxyl group, for PPEylation, was activated with phosgene or N,N′disuccinimidyl carbonate (DSC) and subsequently reacted with N‐Hydroxysuccinimide. The activated chain end was then reacted to proteins via formation of a carbamate linkage with lysine residues.

In this recollection of our work on the polymer–protein conjugate, we explore the biophysical characterization of protein–polymer conjugates, focusing on the impact of *degree of polymerization* (DPn) and *surface grafting density*. We report and resume our finding on how the polymer structurally organizes itself around the protein, its influence on protein folding, and the role of protein size and secondary structure in these processes. These factors collectively influence the conformational changes, interactions, and overall behavior of protein–polymer conjugates, providing valuable insights for optimizing their design and therapeutic applications. Furthermore, we investigate the potential benefits of partial hydrogen‐deuterium substitution in drug development, notably in improving pharmacokinetics and reducing toxicity (DeWitt and Maryanoff 2018; Di Martino et al. 2023; Pirali et al. 2019). While previous studies have primarily focused on the biochemical properties of protein–polymer conjugates, such as their stability, solubility, and immunogenicity, this manuscript delves deeper into their structural and dynamic behavior. In particular, neutron scattering techniques offer unique advantages in elucidating molecular interactions at the atomic level. By combining neutron scattering with isotopic labeling, researchers can precisely probe the structural changes, conformational dynamics, and intermolecular interactions within protein–polymer conjugates. This comprehensive analysis aims to gain a more nuanced understanding of how these conjugates behave at the molecular level, including their interactions with biological macromolecules and their stability under various conditions. Such insights are also crucial for optimizing the design of protein–polymer conjugates for drug development, ensuring their efficacy, safety, and therapeutic potential.

**FIGURE 2:**
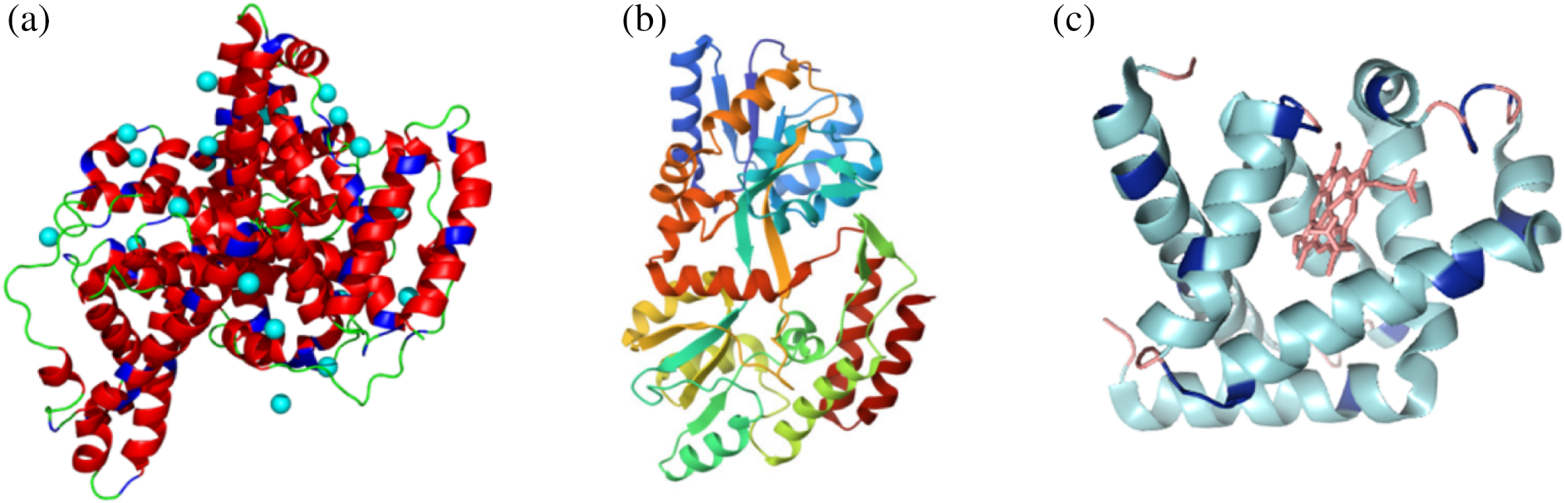
(a) Bovine serum albumin, BSA 583 amino acids; (b) maltose‐binding protein, MBP 392 amino acids; (c) myoglobin, Mb 152 amino acids.

Three distinct proteins, bovine serum albumin (BSA), maltose‐binding protein (MBP), and myoglobin (Mb), will be discussed in this review. These proteins differ in size and structure (Figure 2), with BSA being the largest at 66 kDa, followed by MBP at 42 kDa and myoglobin at 16.7 kDa. Each protein was conjugated with varying numbers of PPEs chains of different molar mass (5 and 10 kDa). The conjugation ratios for MBP ranged from approximately 2–3 polymer chains per protein, while BSA and myoglobin were conjugated with 5, 10, and 20 chains. By comparing the properties of these conjugates, we aimed to elucidate the influence of protein size, polymer molecular weight, and conjugation density on protein folding, stability, and activity.

### 1.1 Neutron scattering technique

Our study of the structure and dynamics of protein–polymer complexes is conducted using neutron scattering as a powerful technique capable of providing detailed structural and dynamic information on complex samples at atomic and molecular scales. Since neutrons interact with atomic nuclei, isotopic substitution—such as hydrogen/deuterium (H/D) in organic compounds—enables selective contrast, allowing researchers to distinguish specific molecular regions through targeted deuteration. The instruments used in this study allow neutron scattering to cover a spatial range of 1 to 1000 Å and a temporal range of 0.1–100 ns (Zaccai 2012).

In this study, the structural properties are analyzed using small‐angle neutron scattering (SANS). The scattering geometry spans a momentum transfer range of *Q* = (4*π*/*λ*)sin(*θ*/2), where *λ* is the neutron wavelength and *θ* is the scattering angle, ranging from 10^−3^ to 0.4 Å^−1^. The scattering contrast between different atomic compositions in the sample—such as hydrogen‐rich versus deuterium‐rich domains—provides crucial structural insights such as interactions, low‐resolution structure, local organization, and shape of the macromolecule. SANS technique has revealed the protein–polymer conformation, including the preferential polymer arrangement around the protein. The dynamic properties are here investigated using the backscattering technique, which measures the temporal autocorrelation of hydrogen atoms. This is represented as exp(−*t*/*τ*), where *τ* is a characteristic timescale in the nanosecond range. This technique analyzes signals arising from self‐diffusion, which involve relatively small energy exchanges and are referred to as elastic or quasi‐elastic neutron scattering (QENS). The scattering is largely dominated by the signal from hydrogen atoms. In practice, for any hydrogenated compound, elastic and QENS neutron scattering provide insights into the dynamics of hydrogen atoms. In this study, elastic scattering refers to the intensity arising from atoms that remain immobile within the timescale defined by the instrument's resolution. This provides quantitative information on the fraction of “immobile” atoms. Since hydrogen has a significantly larger cross‐section, elastic scans as a function of temperature primarily reflect the behavior of hydrogen atoms, allowing us to track how this immobile population evolves with temperature. QENS, on the other hand, measures the relaxation times of proton motions, providing insights into the dynamic behavior of the system.

### 1.2 Structural characterization of protein–polymer conjugates

The variation of polymer architecture and degree of polymerization significantly influences the release kinetics and bioavailability of protein–polymer conjugates. When multiple copies of a polymer are conjugated to the protein surface, it often results in the formation of core‐shell protein–polymer architectures, where the protein core is surrounded by a polymeric shell. This architectural arrangement protects the protein, providing steric shielding, which reduces proteolytic degradation and immunogenicity. It can modulate the rate of protein release and bioavailability, its interaction with biological molecules reducing aggregation, and its overall stability and efficacy. Neutron scattering experiments revealed that these structures are influenced by the polymer's molecular weight and grafting density, and that a balance must be achieved in order do not compromise the protein properties. The combined effects of polymer molecular weight and grafting density can lead to complex structural changes in protein–polymer conjugates. For example, a high molecular weight polymer with a low grafting density might create a loose, flexible shell around the protein, while a low molecular weight polymer with a high grafting density could result in a dense, rigid coating. High degree of polymerization can exhibit greater flexibility, allowing for more conformational freedom and potentially affecting the protein's secondary and tertiary structure. Understanding the interplay between polymer molecular weight and grafting density is fundamental for designing protein–polymer conjugates with desired properties, such as enhanced stability, solubility, and biological activity.

### 1.3 Role of deuteration and polymer molecular weight on conjugate properties

In recent years, there has been a growing interest in the partial substitution of hydrogen by deuterium in drug development since it can impact the protein's structural features, including hydrogen bonds and the packing and orientation of molecules in hydrophobic regions. This substitution has potential benefits, notably in improving the pharmacokinetics and reducing the toxicity of drugs, thereby minimizing adverse effects. Deuterium atoms are heavier than hydrogen atoms; their presence can subtly influence the overall shape and flexibility of molecules. Deuteration can alter the vibrational, rotational, and translational dynamics of molecules, which in turn affects conformational stability. For all those reasons, it has been interesting to investigate the impact of isotopic substitution in the protein conjugation. Perdeuterated MBP (MBP‐D) was purified at the ILL Deuteration Laboratory (Grenoble, France) following the methods that have been developed for the efficient production of deuterium‐labeled samples for a wide range of neutron scattering applications as described by Hartelin and coworkers (Haertlein et al. 2016). Partially hydrogenated polymer–protein conjugates have been created following the procedure described above (Steinbach et al. 2017; Steinbach and Wurm 2015; Steinbach and Wurm 2016).

**FIGURE 3:**
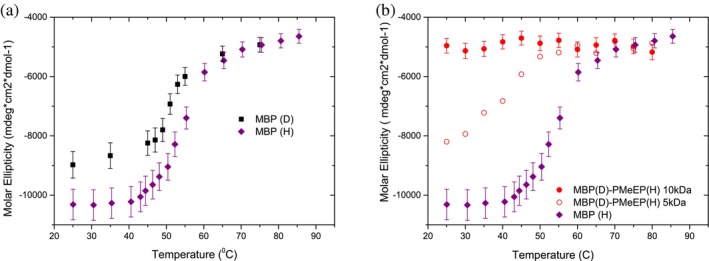
The CD molar ellipticity at 222 nm, a marker of α‐helical content, is plotted as a function of temperature for (a) fully hydrogenated and completely deuterated MBP protein, and (b) partially deuterated MBP conjugates with 5 and 10 kDa polymers. The fully deuterated MBP protein shows a slight destabilization of α‐helical structure, which becomes more pronounced in the polymer conjugates, reflecting the impact of both conjugation and deuteration on protein stability (Russo et al. 2024). Reprinted from Haertlein et al. (2016).

Focusing our investigation on the fully deuterated maltose‐binding protein (Russo et al. 2024), we observed that deuteration impacts its hydrogen bonding networks and hydrophobic interactions, affecting the protein's secondary structure and leading to reduced thermal stability in the proteins. This effect persists during the conjugation process and becomes more pronounced when paired with a high degree of polymerization, resulting in complete unfolding even at room temperature (Figure 3).

The unfolding of the conjugated with the fully deuterated MBP is likely influenced by the combined effects of conjugation‐induced destabilization and deuteration‐induced alterations. Since we lack records for the behavior of other deuterated proteins in the conjugation field context, it is impossible to infer a general rule; however, our observations underscore a relationship between isotopic composition, molecular interactions, and thermal stability in the context of protein conjugation with high‐mass polymers. These findings have implications for the design and optimization of protein–polymer conjugates for various applications. We highlight the importance of protein size and secondary and tertiary structure and deuteration in conjugates, paving the basis *for the design with tailored properties* including solvent‐free destabilization and partially unfolded protein at room temperature.

### 1.4 Comparative analysis of protein types: Protein size and secondary structure

The connection between protein *rigidity*, *secondary structure*, and *protein size* influences protein stability upon conjugation and during the unfolding process. Comparing the circular dichroism (CD) spectra of the three proteins, MBP (Russo et al. 2024), BSA (Russo et al. 2019a), and Mb (Russo et al. 2021), reveals that larger proteins like BSA, with a more rigid structure, retain greater stability upon conjugation than smaller, more flexible proteins like MBP and Mb. While myoglobin shows a decline in conjugate stability at temperatures beyond its native range, MBP—due to its two‐domain structure—displays changes in stability even at room temperature (decrease of the alpha helix structural component), particularly in its deuterated form, as we previously discussed. In both cases, we observe a decrease in the unfolding temperature transition. Furthermore, we note that BSA's secondary structure remains unaffected by variations in polymer grafting density (5, 10, 20 polymers), unlike the smaller myoglobin. On the other hand, MBP, as an example of a two‐domain protein, is affected by conjugation even at low grafting densities. However, increasing the polymer's molecular weight does not further impact MBP's secondary structure, as the polymer distributes across the available protein surface.

In general, it appears that the impact of the polymer grafting on the proteins surface really depends on the intrinsic stability of the chosen protein. Our finding from fluorescence emission also highlights that the de‐structuration mechanism generally begins at the level of the secondary structures, with an impact on the local tertiary structure depending on the polymer shielding on the protein surface (related to the tryptophan structural position).

**FIGURE 4:**
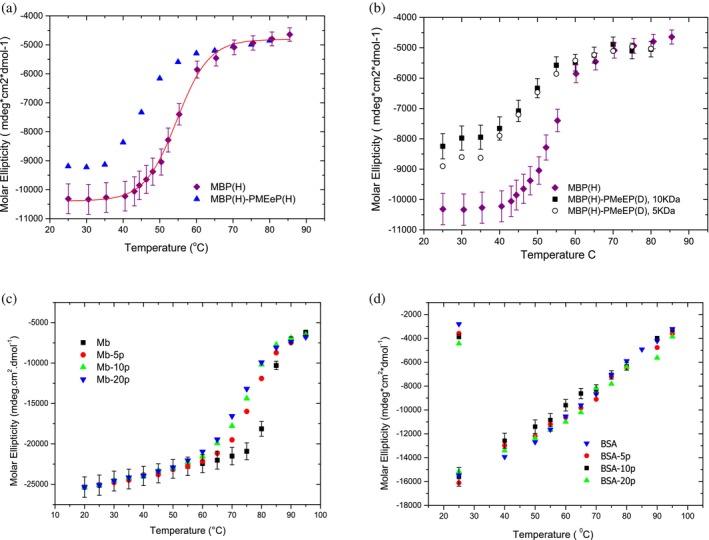
The CD molar ellipticity at 222 nm, indicative of α‐helical content, is plotted as a function of temperature for MBP, BSA, myoglobin (Mb), and their conjugates with the PMEeP‐5kDa polymer. (a, b) MBP, fully hydrogenated and partially deuterated (polymer) conjugates show significant secondary structural changes at room temperature, indicating a loss of α‐helicity upon polymer conjugation. This suggests structural destabilization even under mild conditions. (c, d) Myoglobin and BSA conjugates demonstrate higher thermal stability compared to the native proteins, with the polymer contributing to resistance against unfolding and preserving α‐helical structure at elevated temperatures (Russo et al. 2019a; Russo et al. 2021; Russo et al. 2024). Reprinted with permission from Russo et al. (2024).

Figure 4 reports the molar ellipticity data as a function of temperature for the three proteins and their conjugates.

### 1.5 Structural properties of conjugates in solution

The insights provided by small‐angle neutron scattering (SANS) are especially valuable due to its ability to probe molecular structures at low resolution, making it a critical tool in studying macromolecular assemblies relevant to fields like drug delivery, biomaterials development, and biophysics (Grillo 2008; Liu et al. 2022). SANS was employed to study the low‐resolution structure of protein–polymer conjugates, providing detailed insights into the spatial arrangement of these complex systems on a nanometer scale. This technique was necessary to characterize the overall shape of the protein, its folding, and the surrounding polymer corona, enabling a deeper understanding of the protein–polymer interactions and their impact on thermal stability.

**FIGURE 5:**
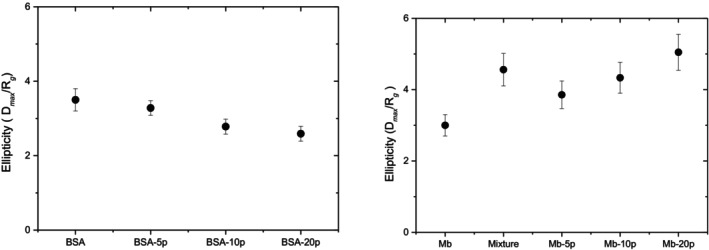
The ratio *D*_max_/*R*_*g*_, derived from the *P*(*r*) analysis, is used to define the ellipticity of the particles. The ellipticity is shown for (a) BSA (Reprinted with permission from Russo et al. (2024)) and (b) myoglobin, along with their respective conjugates, as a function of grafted polymer size and type. This highlights the influence of polymer conjugation on the shape and structural compactness of the protein particles (Russo et al. 2019a; Russo et al. 2024). Reprinted from Russo et al. (2019a).

The analysis of the scattering profile using the pair‐distance distribution function, *P*(*r*), demonstrates that the structural and conformational properties of protein–polymer conjugates are influenced by the protein's intrinsic shape, size, and the grafting density of the polymer chains. In general, increasing the grafting density and polymer molecular weight leads to an expansion in the conjugate's size, as shown by increases in *R*_*g*_ (radius of gyration) and *D*_max_ (maximum dimension). Conjugates of proteins with elongated shapes, such as BSA, tend to evolve toward spherical structures as polymer chains wrap around them. Meanwhile, globular proteins like myoglobin retain their original shape with only minor conformational changes (Figure 5).

**FIGURE 6:**
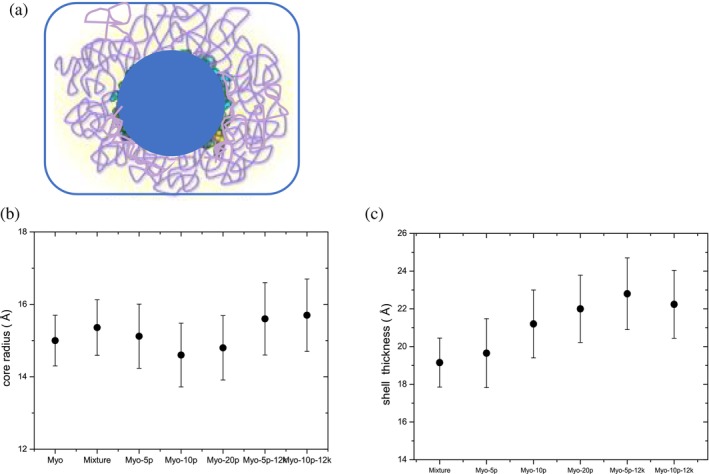
(a) Schematic core‐shell representation, where the protein forms the core and the surrounding polymer, along with associated water molecules, constitutes the shell. (b, c) Small‐angle neutron scattering results fitted using the core‐shell model: (b) core radius, representing the protein size, and (c) shell thickness, representing the polymer layer and associated water molecules. Data correspond to myoglobin conjugates (Russo et al. 2021). Reprinted from Russo et al. (2019a).

The *P*(*r*) profiles consistently reveal a compact protein core surrounded by polymer chains, suggesting elongated or loosely bound chains around the protein (Russo et al. 2019a; Russo et al. 2021; Russo et al. 2024). This captures the interplay between the protein structure and polymer arrangement. To assess the structural features of the conjugates and the polymer distribution around the protein, the analysis was guided by reductionist geometric models (Breßler et al. 2015; Butler et al. 2012). These models describe the conjugates as either elliptical or spherical core‐shell structures, where the protein forms the core and the surrounding polymer constitutes the shell within the solvent. This approach provides complementary perspectives on the preferential polymer arrangement around the protein, clarifying the conformation and stability of the conjugates. Using the core‐shell models, we studied how the size of the protein core changes after polymer grafting and evaluated the thickness and scattering length of the polymer shell, representing the polymer's structural configuration (Figure 6). Combined with CD and fluorescence spectroscopy, these observations confirm that the protein retains its native‐like folding, similar to the native form, at room temperature. Thus, the inferred shell thickness models the polymer distribution on the protein surface, defining the layer where the polymers interact with each other and are attached to the protein–water interface. As a first approximation, this model can be applied to all conjugates. In general, the shell thickness increases with water penetration and polymer extension, both of which are influenced by the grafting density and polymer molecular weight.

**FIGURE 7:**
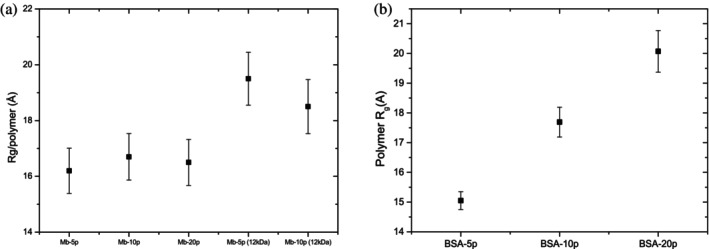
Radius of gyration (*R*_*g*_) of the polymer molecules grafted to the protein, as determined from the Pedersen model for a sphere with attached Gaussian chains. (a) Data for myoglobin conjugates (Reprinted from Russo et al. (2019a)). (b) Data for BSA conjugates (Reprinted with permission from Russo et al. (2024)). For reference, the radius of gyration of the bulk polymer is approximately 32.0 ± 0.3 Å.

Analysis of partially deuterated conjugates (MBP conjugated) allowed us to distinguish contributions from the grafted polymer on the protein and revealed the presence of isolated chains in a Gaussian coil configuration (Russo et al. 2024). The inferred *R*_*g*_ values for the polymer chains, compared to bulk polymers, indicate significant compaction, driven by both the protein chain and the constraints imposed by grafting. This compaction is likely due to portions of the polymer chain adsorbing or interacting with the protein surface, where hydrogen bonds form around the conjugation area, while other segments behave like isolated compact Gaussian coils (Paul et al. 2007). His observation has been shown to hold true for all the conjugates analyzed. Regardless of the protein size, at low grafting densities, a segment of the polymer chain consistently adsorbs to the protein surface due to the likelihood of forming hydrogen bonds around the conjugation area. In the absence of partially deuterated samples, the analysis using the Pedersen model (sphere with attached Gaussian chains) enabled us to estimate the radius of gyration and effective grafting density, along with detecting subtle changes in protein conformation induced by grafting. With increasing grafting density, a gradual shift in polymer conformation from a compact Gaussian coil to an open, star‐like structure was observed (example BSA‐conjugate). At higher densities, steric repulsion between polymer chains leads to further extension, tending toward the bulk polymer dimensions. The protein size significantly affects the ability of polymer chains to adjust on the protein surface, which in turn governs their extension into the surrounding solvent. As a result, grafting polymer chains onto the protein surface also alters the polymer's conformation, regardless of whether the protein is folded or partially unfolded. This suggests that the grafting process itself introduces constraints or interactions that impact the polymer's behavior (Figure 7).

**FIGURE 8:**
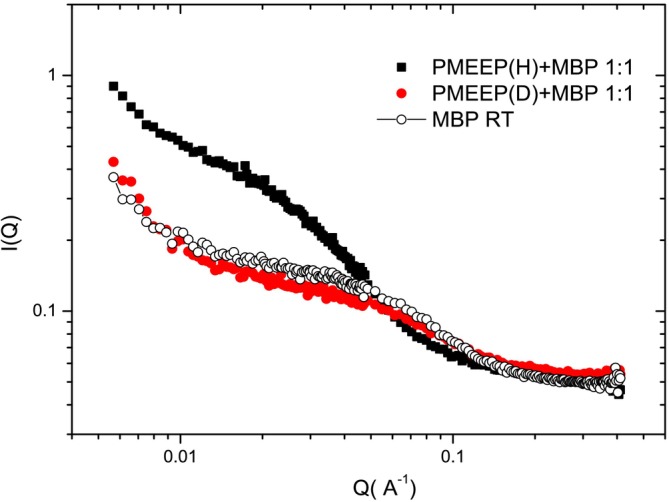
Scattered intensity profiles are compared for the hydrogenated MBP protein, the fully hydrogenated MBP–protein mixture, and the mixture containing the deuterated polymer (PMEEP(D)). Reprinted from Pirali et al. (2019). The analysis shows that the polymer has no significant effect on the globular structure of the MBP protein in the mixtures (Russo et al. 2024).

The investigation of protein–polymer mixtures has been crucial in understanding polymer arrangements on the protein surface and the role of covalent binding in conjugates. Notably, the presence of free polymer in solution does not disrupt the native folding of the protein. Furthermore, when the polymer in the mixture is deuterated, the collected intensity predominantly reflects the protein, producing a spectrum that closely resembles that of the native protein (Figure 8). Additionally, in a mixture, the polymer interacts with the protein surface, as evidenced by the fact that the linear combination of the spectra of the pure polymer and the pure protein does not match the experimentally collected spectrum of the mixture. The fit to the data using a core‐shell model, where the protein radius is fixed, provides an estimate for the thickness of the polymeric shell and the extent of water penetration, indicating a strong interaction and affinity for the protein surface. In comparison to the conjugate, the size of the shell is higher. This difference can be attributed to variations in the number of polymer chains per protein and the presence of grafting constraints, which impose geometric restrictions on the surface. Compared to conjugated polymers, free bulk polymers exhibit greater flexibility in forming hydrogen bonds with the protein. Given the probability of hydrogen bond formation and the polymer's length exceeding the modeled patch size, polymer chains appear to wrap around the protein surface, with a small fraction extending as Gaussian chains adjacent to the protein.

At the unfolding condition, both the protein and the polymer conformations adopt a random coil structures, modeled with a (poly) Gaussian coil model.

## 2 DYNAMICAL PROPERTIES OF CONJUGATES

Proteins are not rigid molecules; they exhibit flexibility at different levels. Protein dynamics fluctuations (small movements of amino acid side chains, loop and domain motions) occur over a broad range of timescales and are fundamental to protein function, enabling critical processes such as structural flexibility, enzymatic activity, and interactions with other biomolecules (Henzler‐Wildman and Kern 2007; Parak 2003). Factors such as solvation, pressure, and molecular interactions—including complexation and conjugation—modulate these motions and, by extension, biological activity. Using elastic and quasi‐elastic neutron scattering techniques, we aim to investigate the effects of polymer conjugation on protein dynamical fluctuations in the *ps* timescale (local diffusion and side‐chain rotations). Using partially deuterated conjugate systems, we are able to disentangle the polymer dynamics from the protein dynamics and meaningfully address the coupling between both components. Central questions include whether polymer coatings can mimic the role of hydration water in promoting thermal structural fluctuations, how polymer dynamics contribute to protein activity, and the factors that determine the stability of polymer–protein conjugates. How do polymers conformation around protein surfaces impact the protein local dynamic activity? Addressing these questions will shed light on the structure–function–dynamics relationship in polymer–protein conjugates and help identify the governing principles of their stability and performance. Identifying the common parameters that govern stability and flexibility in these systems, we aim to establish a foundation for the rational design of polymer–protein conjugates with enhanced functionality and tailored properties. The structure and dynamics of polymer–protein conjugates are inherently governed by the characteristics of both components. Protein size, shape, and intrinsic dynamics must be considered alongside polymer properties such as chain length, hydrophilicity, and degradation behavior.

Through our investigation of three different proteins, BSA (Russo et al. 2019b), MBP (Russo et al. 2016), and Mb (Russo et al. 2020), we demonstrate that the polymer, being dynamically active at the protein interface, plays a crucial role in modulating the protein fluctuations. This interaction significantly enhances the magnitude of protein dynamics, underscoring the importance of polymer–protein interactions in influencing protein behavior. Notably, our findings reveal that polymer modification can, to some extent, mimic the effect of hydration water in enhancing protein dynamics, underscoring its potential as a functional substitute in specific contexts.

**FIGURE 9:**
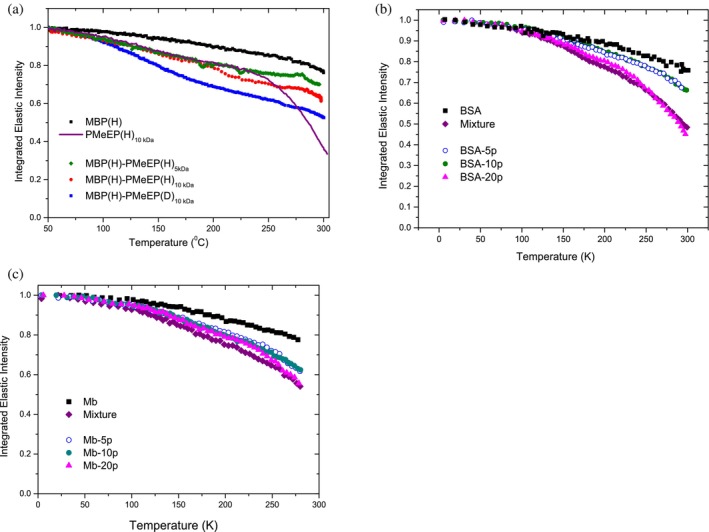
Dry state: the effect of conjugation on the dynamics. The elastic intensity probed in an elastic neutron scattering experiment arises from atoms, which are immobile on the timescale of the instrumental resolution. Elastic scans give access to the transition temperatures and mean square displacements. The figures report integrated elastic intensity as a function of temperature for dry conjugates and their components for MBP, BSA, and Mb protein. (a) MBP conjugates have about four grafted polymers. The polymer glass transition can be observed at around 240 K. A smooth and linear decrease of the intensity as a function of temperature is characteristic for the native hydrogenated MBP protein, the MBP(H)‐PMeEP(H)_5kDa_ (fully hydrogenated sample, polymer MW = 5 kDa), the MBP(H)‐PMeEP(H)_10kDa_ (fully hydrogenated sample, polymer MW = 10 kDa), and the MBP(H)‐PMeEP(D)_10kDa_ (partially deuterated sample, enhancing the protein dynamics). We observe a lack of abrupt dynamical transition and that increasing the polymer molecular weight decreases the elastic intensity as a signature of increased flexibility and fast dynamics (Russo et al. 2016). Reprinted with permission from Russo et al. (2019b). (b) BSA grafted with different numbers (5–20) of polymer chains (5 kDa) compared to those in dry BSA and dry mixtures. Dynamic enhancement increases with the number of grafted chains and in the mixture (Russo et al. 2019b). Reprinted with permission from Henzler‐Wildman and Kern (2007). (c) Mb grafted with different numbers (5–20) of polymer chains (5 kDa) compared to those in dry Mb. In accordance with the previous figure, we observe a decrease in the elastic intensity. Compared to the BSA, we remark a different temperature dependence, most likely related to a distinct polymer conformation on the protein surface (Russo et al. 2020). Reprinted from Russo et al. (2016).

**FIGURE 10:**
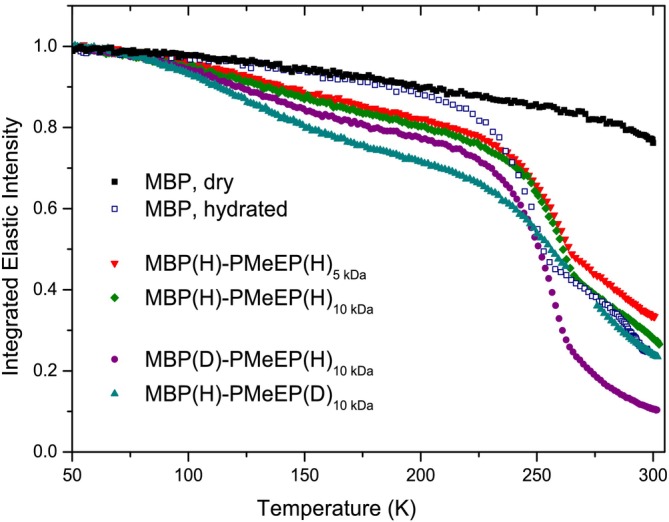
Hydrated state: the effect of conjugation on the dynamics Integrated elastic intensity as a function of temperature for hydrated conjugates compared to dry and hydrated native MBP. Reprinted with permission from Russo et al. (2019b). All hydrated curves show an abrupt change in the elastic intensity as a fingerprint of dynamical transition. That is, the larger the loss of elastic intensity the higher is the flexibility of the system. The dynamical transition of hydrated MBP is observed at *T* = 200 K while for all other samples, given the presence of the polymer, it is observed at *T* > 225 K.

For all investigated protein conjugates, in the dry state, we observed that protein exhibits increased flexibility compared to its native form, consistent with the influence of the polymer solvation. Despite this enhanced flexibility, at low grafting density and low molecular weight (investigation on the MBP, Mb, BSA conjugates with 5 and 10 grafted polymers at 5 kDa), we observe no evidence of a dynamic transition, as is typical behavior of hydrated proteins. Additionally, the absence of a polymer‐glass transition (physical transition where the polymer changes from hard to soft, in this case *T*_*d*_ 240 K). Figure 9a confirmed the importance of inter‐polymer interactions and fluctuations. A smooth dynamical transition (Doster et al. 1989; Tournier and Smith 2003), of the whole complex, appears around 200 K, when either grafting density or polymer molecular weight is high (20 grafted polymer or 12 kDa molecular weight) as a signature of an interplay between the protein size, polymer molecular weight, and density at the interface (Figure 9a–c). At both low and high grafting densities, low‐temperature transitions associated with CH_3_ rotation are observed (Perticaroli et al. 2013). Only upon the addition of hydration water does the conjugate undergo a better‐defined dynamic transition aligning with the typical behavior of hydrated proteins (Figure 10). Our finding suggests that below a certain threshold, polymer solvation alone is insufficient to unfreeze fluctuations on the picosecond–nanosecond timescale in biomolecules, as widely observed in the context of protein hydration. Additionally, we reveal that water molecules preferentially hydrate the hydrophilic polymer chains rather than the protein, which results in reduced hydration of the protein surface, increased mobility of the polymer chains, and a shift in the dynamic transition temperature to higher values. Depending on their conformation and local crowding, polymers on the protein surface induce confinement dynamics. The attached polymer creates **steric hindrance** around the protein, reducing its available conformational space. The grafted polymer creates a **local crowded environment**, mimicking the effects of macromolecular crowding in cells.

This highlights their role in reducing the accessible geometrical space available to the protein and restricting its diffusion to localized fluctuations.

For both BSA and Mb protein, we observed that, at equivalent polymer content, the mixtures and conjugates exhibit distinct dynamical behavior. Comparing protein–polymer mixtures to proteins with covalently attached polymers reveals how factors such as grafting, molecular weight, and hydrophilicity influence the protein's relaxation dynamics. Notably, the protein dynamics are more pronounced in the protein–polymer mixtures than in either the native protein or the conjugates, even in the dry state. This suggests a fundamentally different coupling between polymer and protein dynamics in the conjugates. The arrangement of polymer chains on the protein surface differs significantly between mixtures and conjugates. In conjugates, the presence of constraint points—arising from covalent grafting—limits the dynamical impact of the polymer on the protein. This structural distinction highlights the role of polymer configuration and interaction in modulating protein behavior, with mixtures offering greater flexibility and enhanced dynamics compared to the more restricted conformations in conjugates.

In conclusion, we observe that the increase in protein dynamics is a general trend, with the possibility that different types of polymers—such as linear, branched, or charged—can influence global dynamics. Like protein hydration (Russo et al. 2010; Russo et al. 2013; Russo and Teixeira 2015), the polymer's ability to enhance dynamics transition and enhancement is contingent upon exceeding a critical threshold. This threshold, influenced by polymer density and molecular weight, underscores the need for a sufficient polymer network to couple effectively with the protein surface.

## 3 CONCLUDING REMARK

Our recollection helps to shed light on the intricate relationship between polymer conjugation and protein dynamics, revealing how factors such as polymer architecture, molecular weight, hydration, and conjugation density collaboratively shape the structural and functional properties of protein–polymer conjugates. Hydration emerges as a cornerstone for preserving and facilitating sharp and well‐defined dynamical transitions. Without adequate hydration, these transitions are smoothed or suppressed, which highlights the intricate dependency of protein function on both its immediate environment and polymer architecture.

The use of deuteration has proven invaluable for probing the fine details of these complexes, offering unprecedented insight into how isotopic substitution influences protein stability and motion. However, the study also highlights a duality in deuteration's role: while it enhances analytical precision, it may introduce destabilizing effects, particularly in conjugates with high molecular weight polymers. We discussed the inherent difficulty of analyzing samples partly deuterated as representative of full hydrogenated samples, if one takes into account that deuteration itself modifies the dynamical behavior. This delicate balance calls for careful consideration when designing experimental conditions or therapeutic formulations that rely on isotopic modifications. Interestingly, larger proteins exhibit greater resilience to these destabilizing factors, suggesting that intrinsic protein properties play a role in modulating the effects of polymer conjugation and deuteration.

From a materials science perspective, the research identifies PPEs as a promising alternative to PEG, with the potential to mitigate safety concerns associated with long‐term PEG usage while maintaining comparable biocompatibility and stability. The tunable properties of PPEs, combined with their degradability, make them ideal candidates for creating safer and more effective bioconjugates for therapeutic applications. By tailoring polymer characteristics such as branching, molecular weight, and hydrophilicity, it is possible to precisely control the dynamic and structural behavior of protein conjugates.

Neutron scattering and complementary spectroscopic techniques have been instrumental in unraveling the complex interplay between polymer properties and protein behavior. These methods not only elucidate how polymer solvation and grafting density influence protein folding and stability but also provide insights into the mechanisms underlying temperature‐dependent dynamic transitions.

Looking ahead, future research should aim to refine the design of protein–polymer conjugates by exploring advanced polymer architectures, including block copolymers, dendritic structures, and stimuli‐responsive materials. This work lays a solid foundation for advancing protein–polymer conjugates as versatile tools in drug delivery and biopharmaceutical development. By striking a balance between protein flexibility and structural integrity, it is possible to unlock the full potential of these bio‐hybrid systems, paving the way for next‐generation therapies with improved efficacy, stability, and safety profiles.

## AUTHOR CONTRIBUTIONS

**Daniela Russo:** Conceptualization; investigation; methodology; validation; writing—review and editing and formal analysis. **Frederik Wurm:** Investigation; writing—review and editing; visualization and methodology. **Jose Teixeira:** Validation and investigation.

## Data Availability

The data that support the findings of this study are available from the corresponding author upon reasonable request.
